# Supplementary material for: Difluoroester solvent toward fast-rate anion-intercalation lithium metal batteries under extreme conditions
Source: Nat Commun. 2024 Jun 26;15:5408. doi: 10.1038/s41467-024-49795-9 (PMC11208432; doi:10.1038/s41467-024-49795-9)
Supplement: Supplementary file 1 — Supplementary Information [file 41467_2024_49795_MOESM1_ESM.pdf]

Difluoroester solvent toward fast-rate anion-intercalation lithium metal batteries under extreme conditions

Supplementary Figures

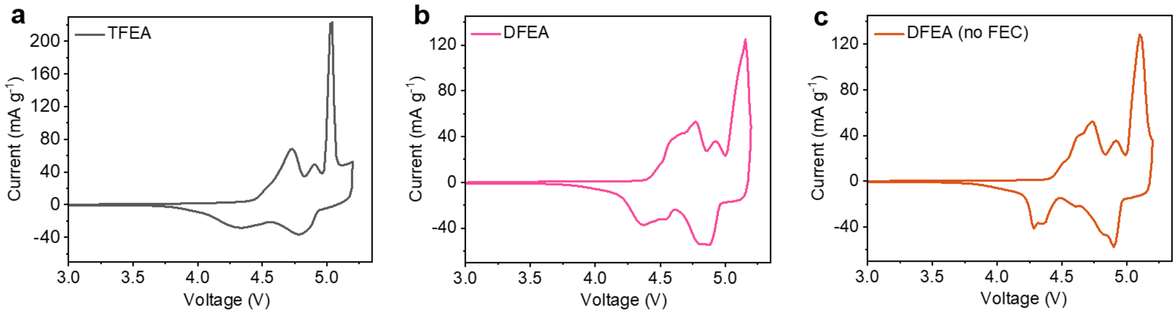

Supplementary Figure 1. CV cures of the anion de-/intercalation from/into graphite cathodes using **a** TFEA, **b** DFEA and **c** DFEA (without FEC)-based electrolytes.

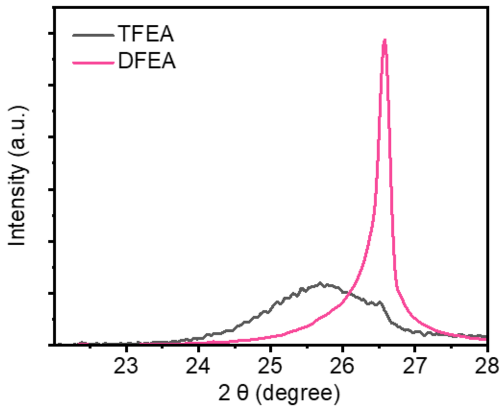

Supplementary Figure 2. XRD pattens of the graphite cathodes using TFEA and DFEA-based electrolytes at the fully discharge state in the 1st cycle at 20 mA g^{-1}.

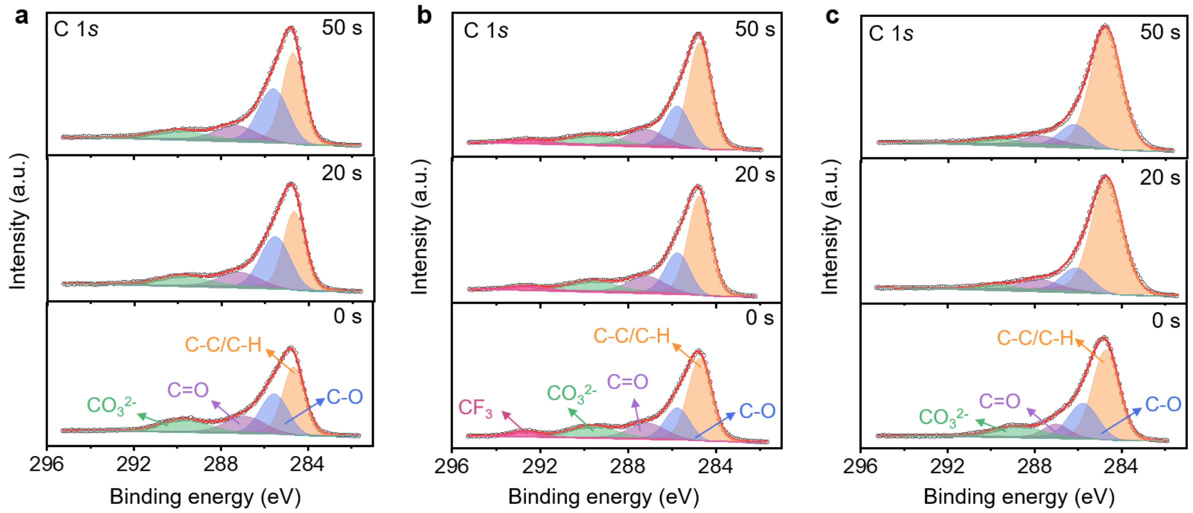

Supplementary Figure 3. C 1*s* XPS depth profiles of the graphite cathodes after the 2nd cycle at the cutoff voltage of 5.2 V, using **a** EA, **b** TFEA and **c** DFEA-based electrolytes. C-C/C-H: 284.7 eV, C-O: 286 eV, C=O: 287 eV, CO_3^{2-} (e.g., $ROCO_2Li$, Li_2CO_3): 289 eV, CF_3: 292.5 eV[1].

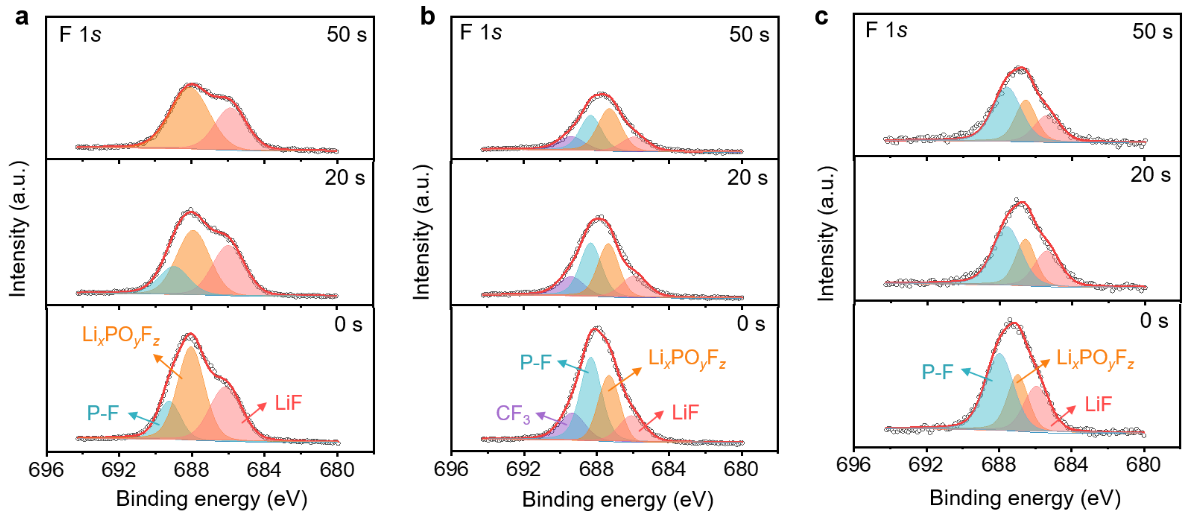

Supplementary Figure 4. F 1*s* XPS depth profiles of the graphite cathodes after the 2nd cycle at the cutoff voltage of 5.2 V, using **a** EA, **b** TFEA and **c** DFEA-based electrolytes. P-F: 688.5 eV[2]; $Li_xPO_yF_z$: 687.8 eV[3]; LiF: 685.5 eV, CF_3: 689 eV[1].

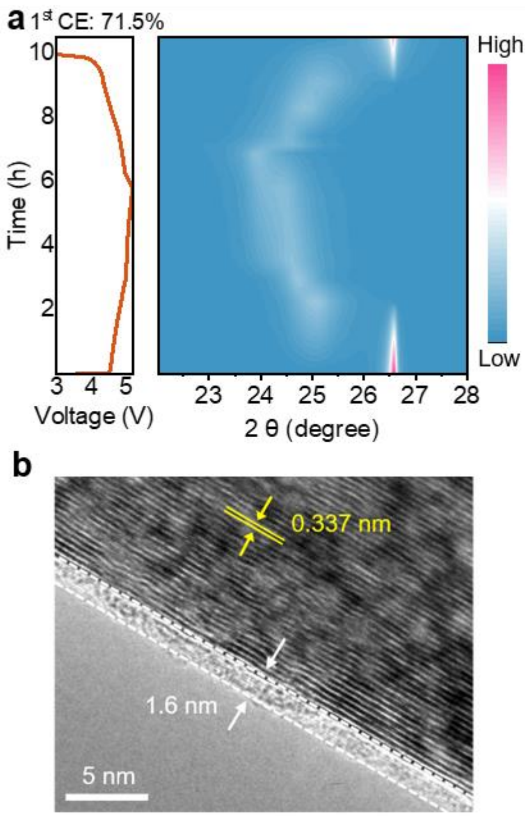

Supplementary Figure 5. a Intensity contour map obtained from the ex-situ XRD patterns of Li||graphite cells during initial charge-discharge processes at 20 mA g^{-1} using FEC-free 1.2 M $LiPF_6$ in DFEA electrolyte. The corresponding voltage-testing time curve is shown on the left panel. **b** TEM image of the graphite cathode after the 1st cycle in FEC-free 1.2 M $LiPF_6$ in DFEA electrolyte.

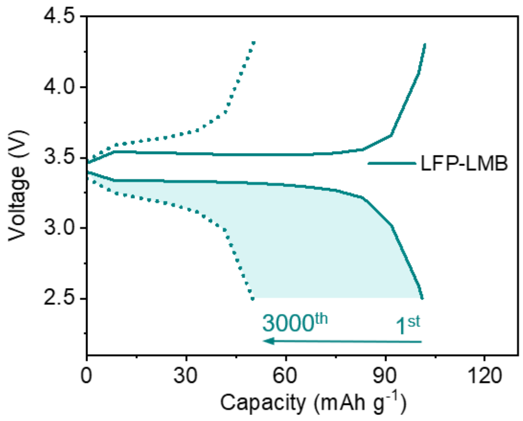

Supplementary Figure 6. Charge-discharge profiles of Li||LFP LMB at the 1st and 3000th cycles at 1 A g^{-1}.

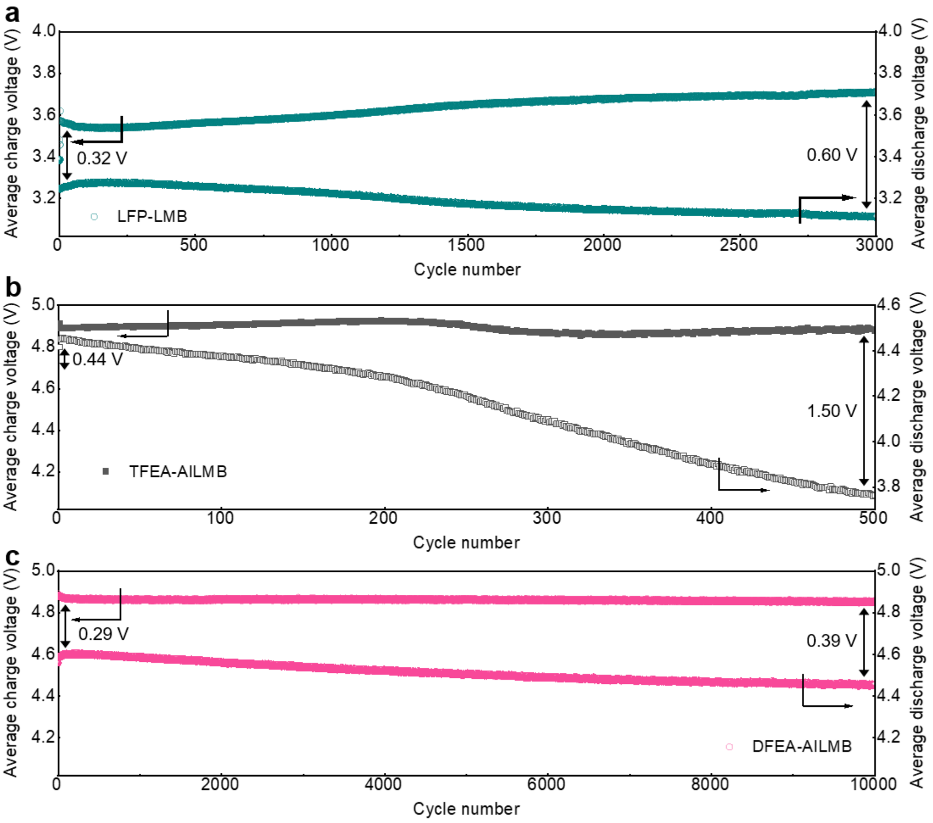

Supplementary Figure 7. Evolution of cell polarizations (the gap between the average charge voltage and the average discharge voltage) during cycling for **a** Li||LFP LMB, and AILMBs using **b** TFEA and **c** DFEA-based electrolytes at 1 A g^{-1}.

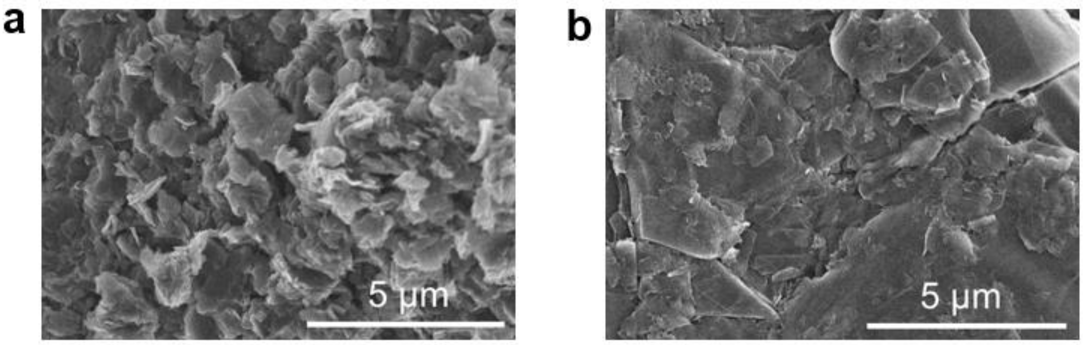

Supplementary Figure 8. SEM images of graphite cathodes after 200 cycles in **a** TFEA and **b** DFEA-based electrolytes at 1 A g^{-1}.

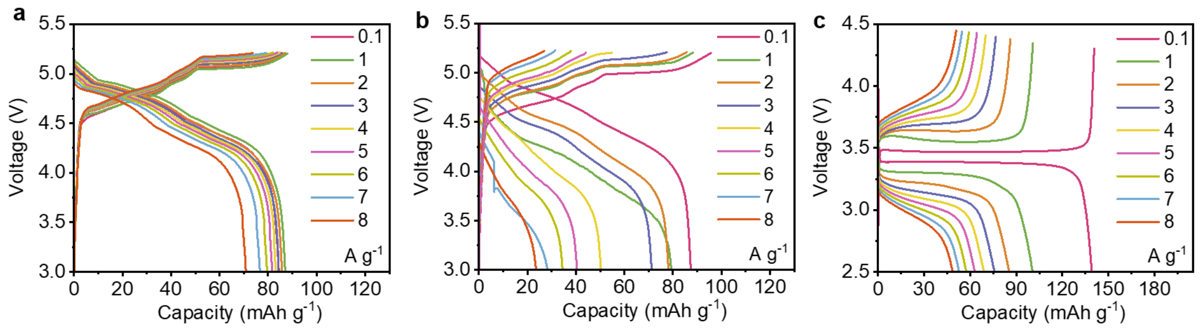

Supplementary Figure 9. Evolution of the voltage curves for AILMBs with **a** DFEA and **b** TFEA-based electrolytes, and for **c** Li||LFP LMB at different current densities.

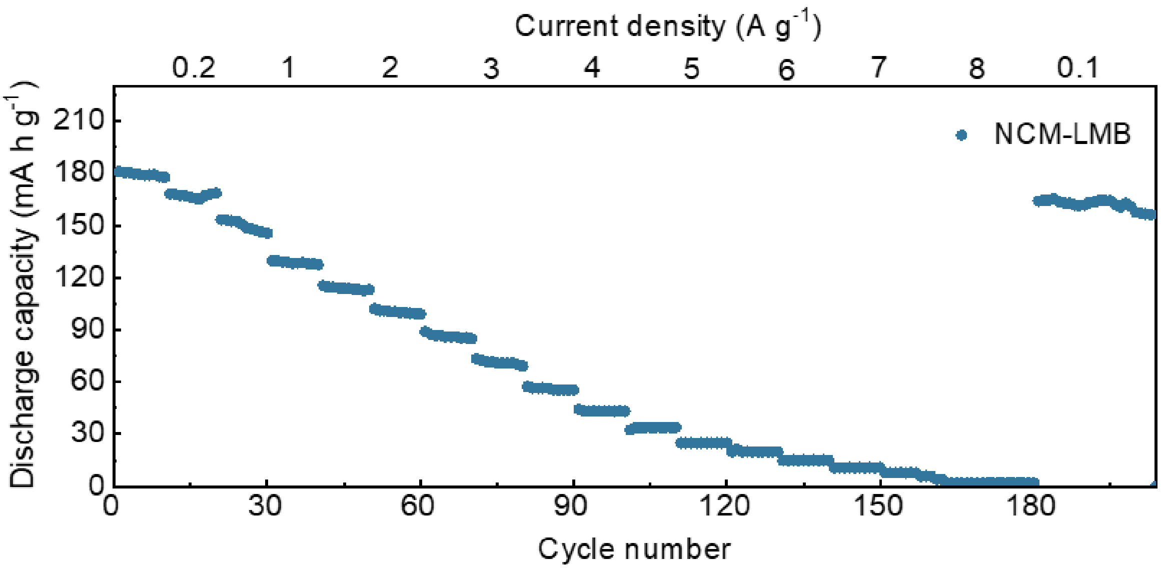

Supplementary Figure 10. Rate performance at various current densities from 100 mA g^{-1} to 8 A g^{-1} for Li||NCM811 LMB.

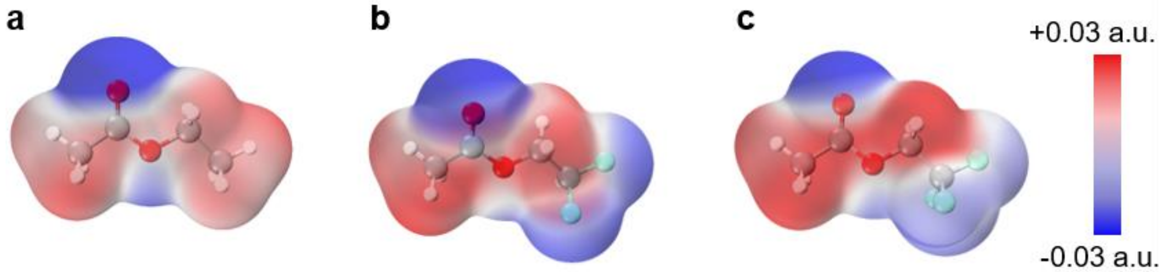

Supplementary Figure 11. ESP maps of EA, DFEA and TFEA solvent molecules. Gray, white, red, and green balls represent carbon, hydrogen, oxygen, and fluorine atoms, respectively.

It is seen that the negative charge interacting with Li^+ is predominately located on the O atoms, particularly the C=O group, on the EA solvent. Meanwhile, the positive charge interacting with PF_6^- anions is primarily concentrated on the $-CH_2$/$-CH_3$ groups (Supplementary Fig. 12a). With increasing degree of fluorination on EA solvent, the change on F atoms become less negative, while the charge on $-CH_2$/$-CH_3$ groups is less positive (Supplementary Fig. 12b, c). This is attributed to the local dipole on the difluoro asymmetric $-CHF_2$, which is absent on the trifluo $-CF_3$ counterpart[4]. The local dipole in the DFEA solvent enhances the Li^+-solvent interaction but weakens the PF_6^--solvent interaction in the electrolyte.

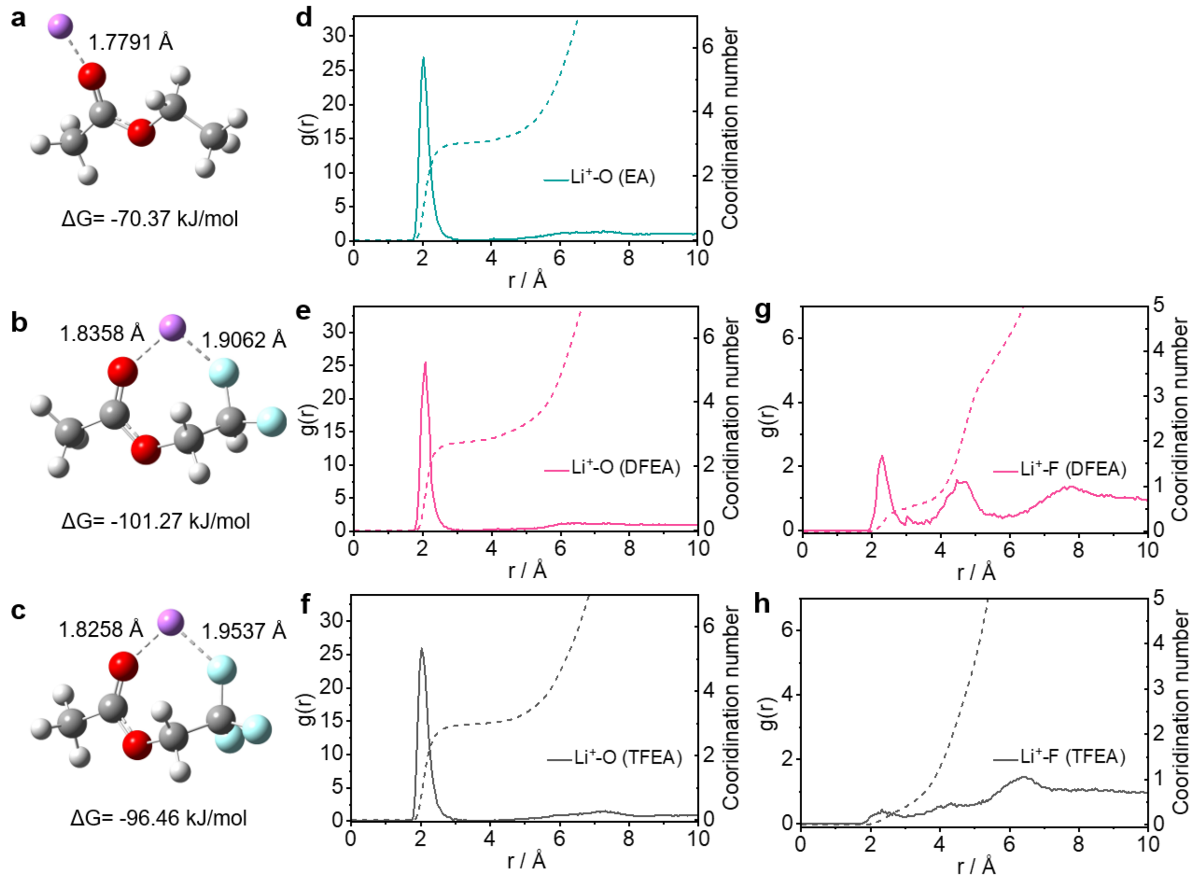

Supplementary Figure 12. Coordination structures and binding energies of **a** Li^+–EA, **b** Li^+–DFEA and **c** Li^+–TFEA. Li^+ RDF obtained from MD simulations of **d** EA, **e, g** DFEA and **f, h** TFEA electrolytes. Solid lines represent $g(r)$ while dashed lines represent coordination number.

The binding configurations and corresponding binding energies (ΔG) between Li^+ and each solvent molecule were shown in Supplementary Fig. 12a-c. It is seen that Li^+ ions form a chelating seven-member ring geometry for DFEA or TFEA whose F atoms also participate in the coordination structure. Moreover, Li^+ exhibits stronger interaction with -CHF_2 than -CF_3, as evidenced from the shorter Li–F distance of 1.906 versus 1.954 Å for -CF_3. Such a stronger interaction between Li^+ and the-CHF_2 can be attributed to the local dipole which is more negatively charged than -CF_3 (Supplementary Fig. 11).

To further investigate the Li^+ solvation sheath, the RDFs and coordination numbers were obtained from MD simulations (Supplementary Fig. 12d-h). It is worth noting that the coordination of Li^+ to F atoms can be seen for DFEA and TFEA electrolytes. Clearly, the Li–

F RDFs displays that more F atoms on $-CHF_2$ participating in Li^+ solvation compared with that on $-CF_3$. These results are consistent with the charge distribution and binding energy in Supplementary Fig. 11 and Supplementary Fig. 12a-c.

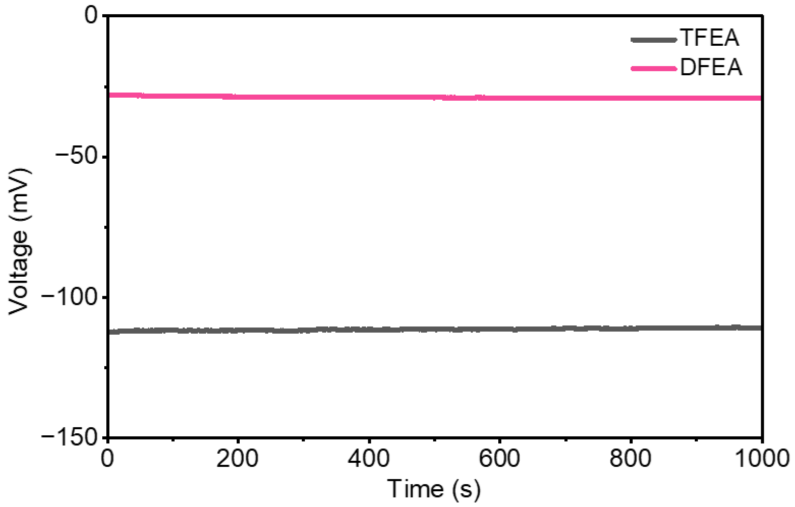

Supplementary Figure 13. Voltage profiles of open-circuit voltage (OCV) measurements (reflecting the solvation energy of electrolytes), where TFEA and DFEA-based electrolytes were measured against a 1 M $LiPF_6$- diethyl carbonate (DEC) reference electrolyte solution. The salt bridge contained 3 M $LiPF_6$-EMC solution.

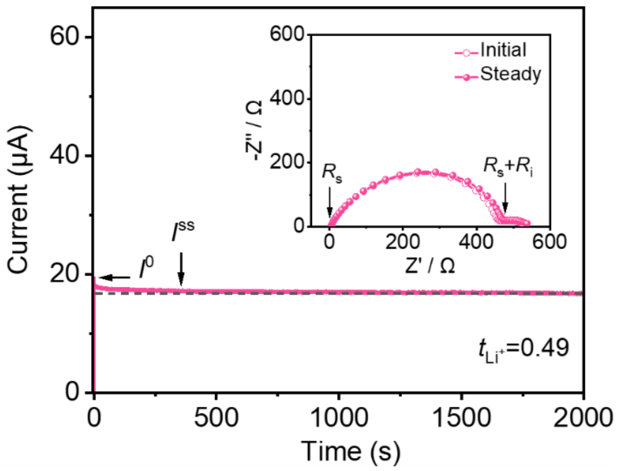

Supplementary Figure 14. Chronoamperometry profile of the Li||Li cells with DFEA-based electrolytes. The applied polarization voltage was 10 mV. The EISs before and after the polarization are shown in the inset, and related parameters are concluded in Supplementary Table 3.

The Li^+ transference number (t_{Li^+}) of the electrolyte was measured using the method described by Abraham et al[5]. Symmetric Li||Li cell was assembled and the polarization currents (the initial (I^0) and steady-state (I^{ss}) current) were recorded under a small polarization potential (ΔV) at 10 mV. Besides, the initial and steady-state EIS measurements before and after the potentiostatic polarization were conducted, obtaining the electrolyte resistances (R_s^0 and R_s^{ss}) and the interfacial resistances (R_i^0 and R_i^{ss}). The t_{Li^+} was calculated based on the following equation[5]:

$$t_{Li^+} = \frac{I^{ss}(\Delta V - I^0 R_i^0)}{I^0(\Delta V - I^{ss} R_i^{ss})} \tag{1}$$

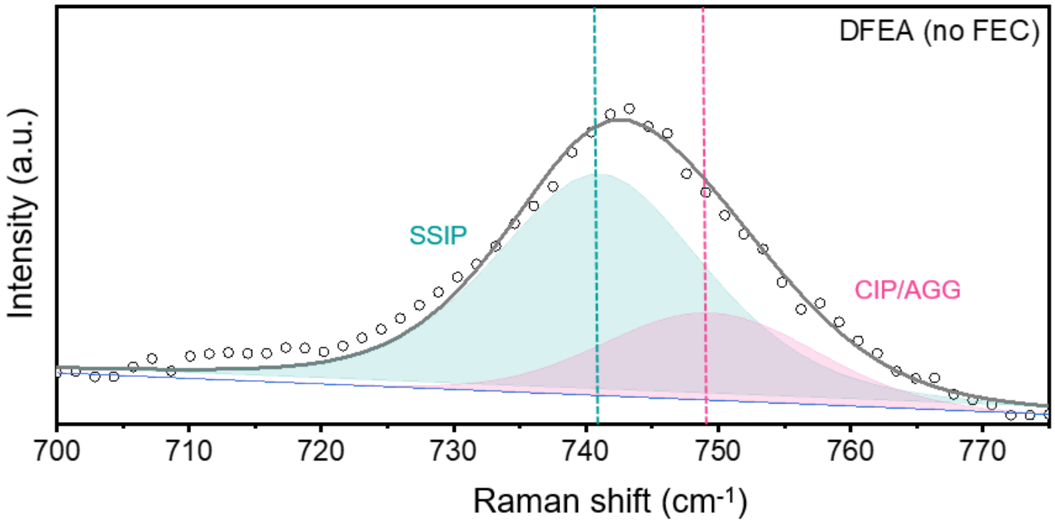

Supplementary Figure 15. Raman spectra of DFEA (without FEC)-based electrolyte.

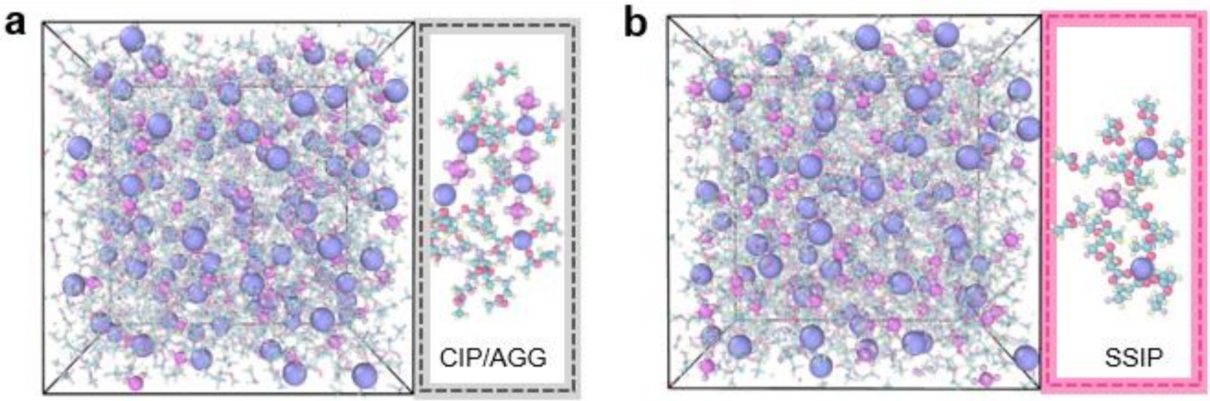

Supplementary Figure 16. Snapshots obtained from MD simulations of **a** TFEA and **b** DFEA-based electrolytes.

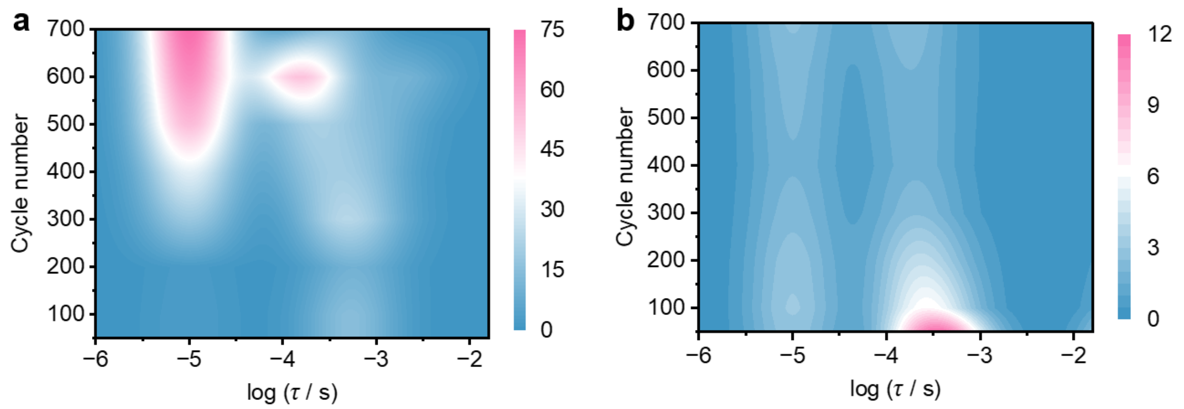

Supplementary Figure 17. In-situ DRT data of Li||Li cells cycling at 0.5 mA cm^{-2} with a cutoff capacity of 0.5 mAh cm^{-2} with **a** EC/DEC and **b** DFEA-based electrolytes.

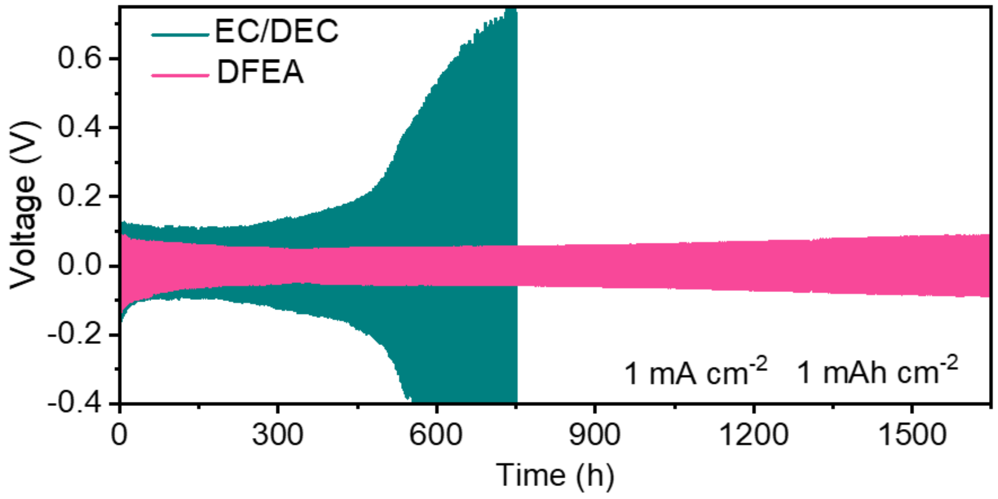

Supplementary Figure 18. Voltage profiles of Li||Li symmetric cells with the EC/DEC and DFEA-based electrolytes at 1 mA cm^{-2} with a cutoff capacity of 1 mAh cm^{-2}.

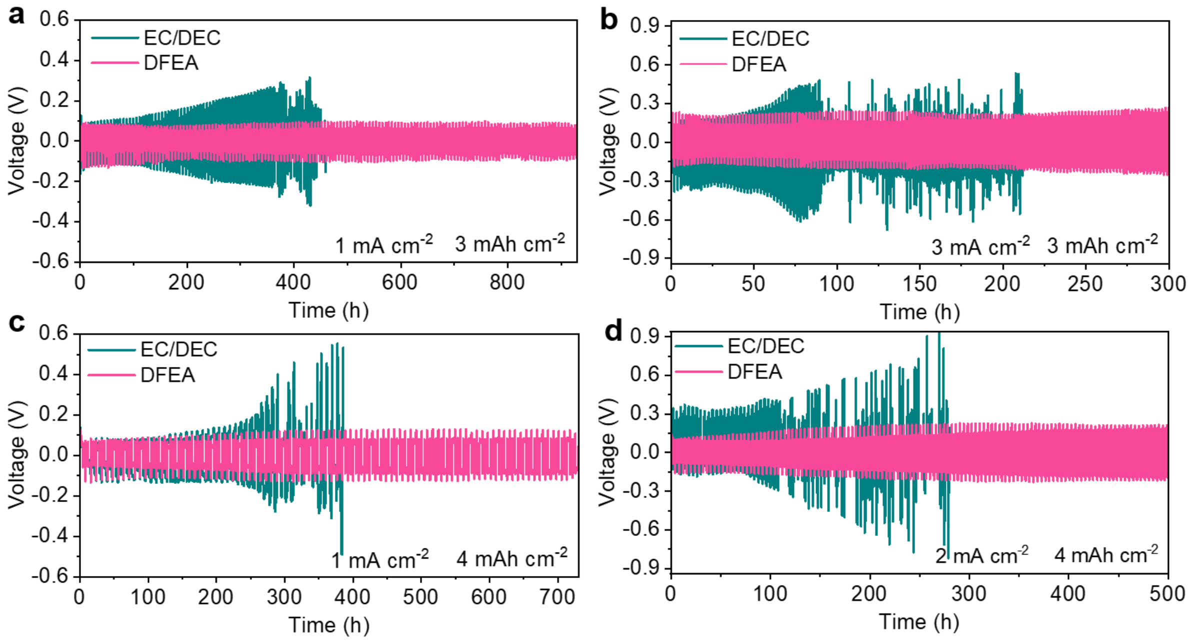

Supplementary Figure 19. Voltage profiles of Li||Li symmetric cells using EC/DEC and DFEA-based electrolytes with a cutoff capacity of 3 mAh cm^{-2} at **a** 1 mA cm^{-2}, **b** 3 mA cm^{-2}, and with an areal capacity of 4 mAh cm^{-2} at **c** 1 mA cm^{-2}, **d** 2 mA cm^{-2}.

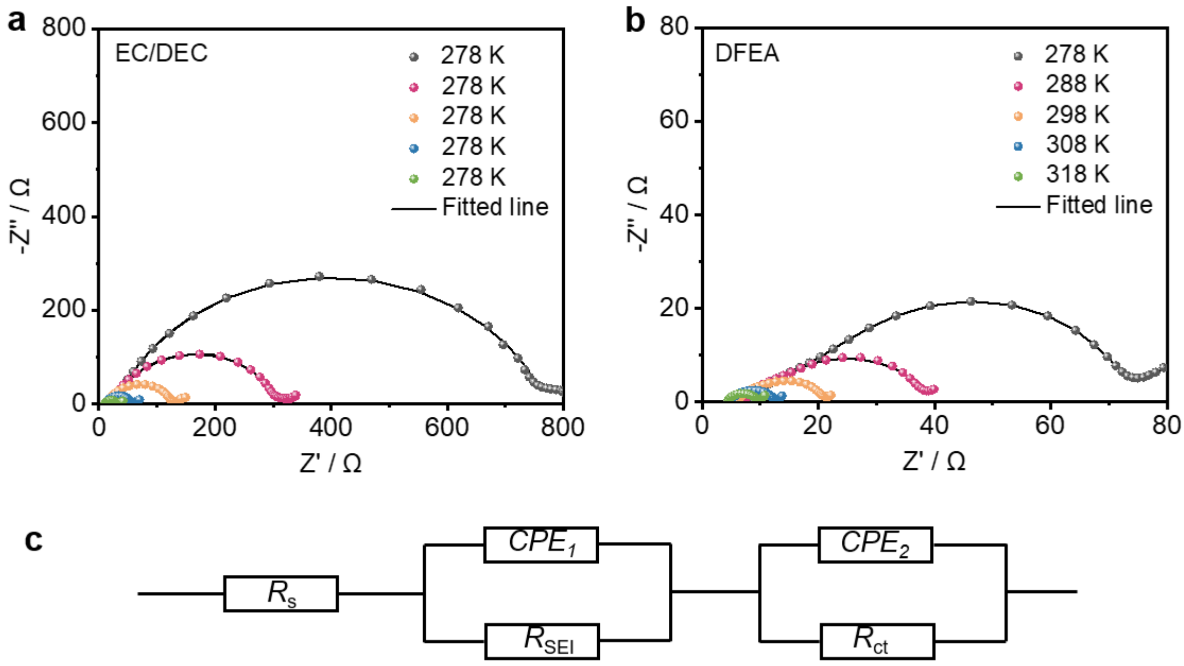

Supplementary Figure 20. Nyquist plots of Li||Li cells using **a** EC/DEC and **b** DFEA-based electrolytes at various temperatures. **c** The equivalent circuit employed to fit the Nyquist plots. The related fitting parameters are presented in **Supplementary Table 5**.

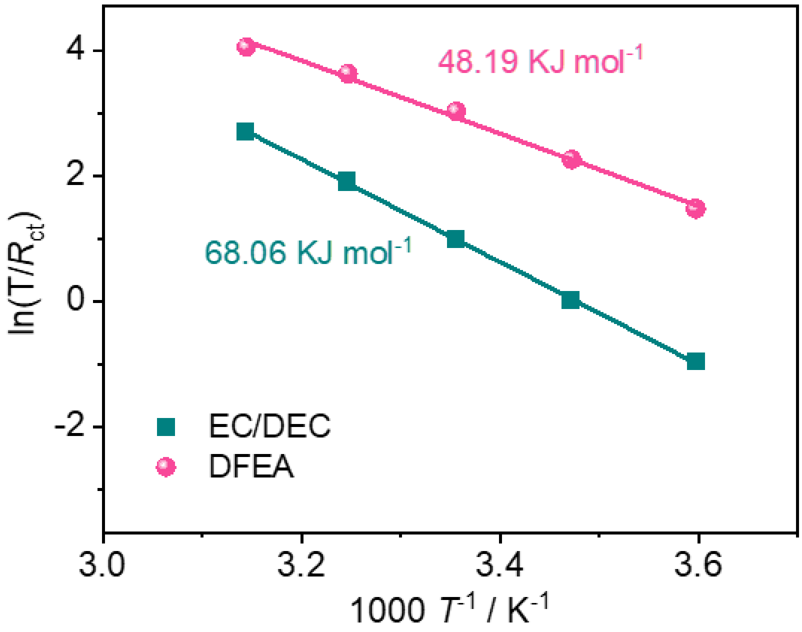

Supplementary Figure 21. Activation energies of R_{ct} derived from Nyquist plots using EC/DEC and DFEA-based electrolytes.

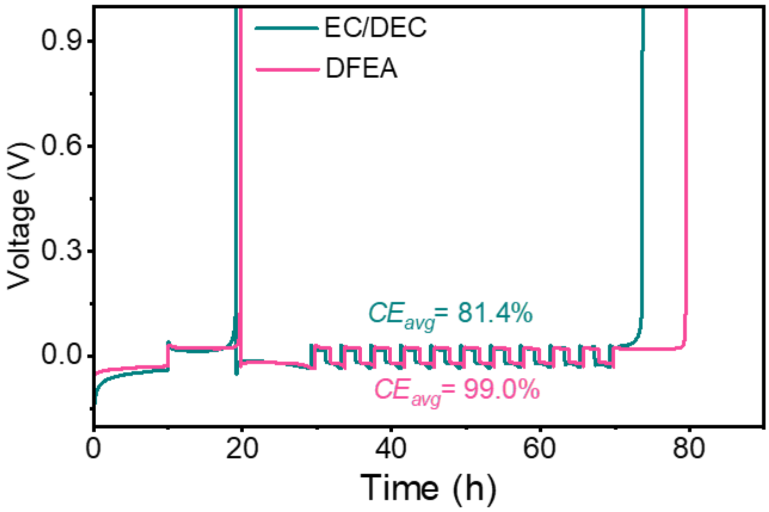

Supplementary Figure 22. CE_{avg} values of Li plating/stripping at 0.5 mA cm^{-2} with a cut-off capacity of 1 mAh cm^{-2} using Li||Cu cells with EC/DEC and DFEA-based electrolytes.

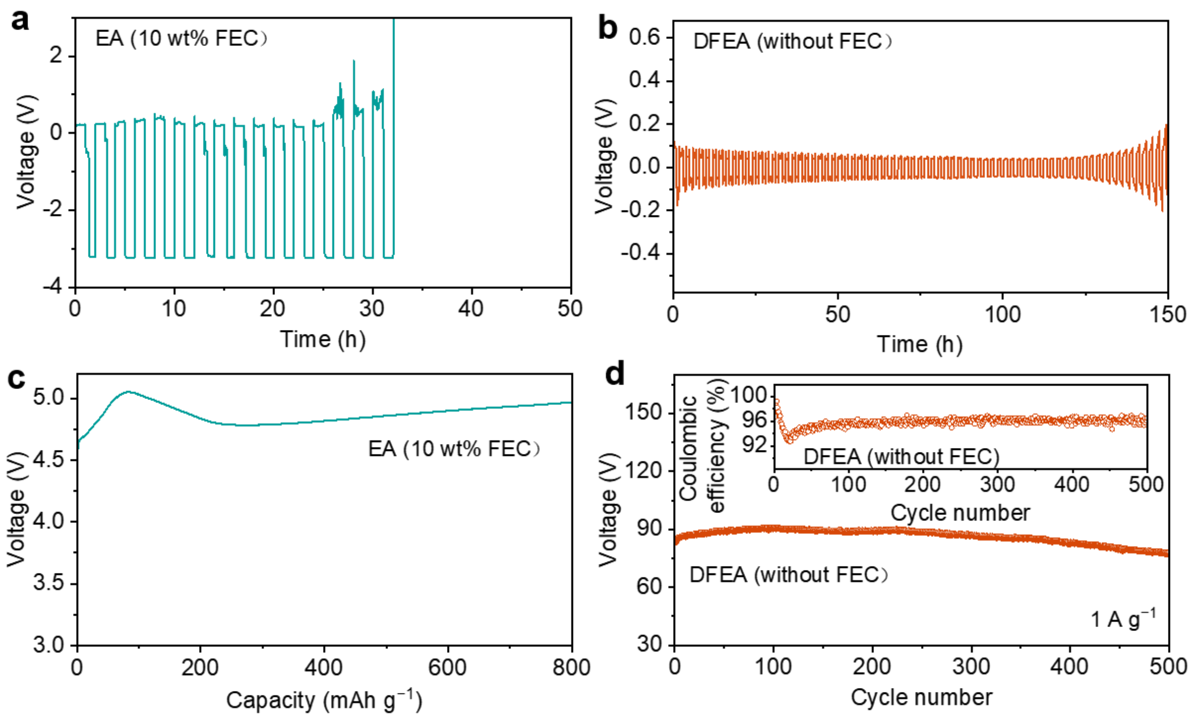

Supplementary Figure 23. Voltage profiles of Li||Li cells using **a** EA (10 wt% FEC) and **b** DFEA (without FEC) electrolyte at 0.5 mA cm^{-2} with a cutoff capacity of 0.5 mAh cm^{-2}. **c** Voltage curves of Li||graphite cells using the EA (10 wt% FEC) electrolyte at 20 mA g^{-1} at 25 °C. **d** Cycling performance of Li||graphite cells using DFEA (without FEC) electrolyte at 1 A g^{-1} after three activation cycles at 20 mA g^{-1} at 25 °C. Inset is the Coulombic efficiency.

The cycling behavior of Li||Li cells based on the DFEA (without FEC) and the EA (with 10 wt% FEC) electrolytes were investigated at 0.5 mA cm^{-2}. It is seen that the EA electrolyte with FEC addition exhibits a large overpotential and quickly suffers from short-circuiting (Supplementary Fig. 23a). In contrast, the cell using DFEA (without FEC) electrolyte shows a smaller overpotential and longer lifespan (Supplementary Fig. 23b). In addition, the FEC addition into the DFEA electrolyte can enable highly reversible Li plating/stripping (Fig. 4a). These results suggest that it is the combined effect of FEC and DFEA that endows the Li||Li cell with long-term cycling stability. Besides, it is seen that AILMBs using EA with 10 wt% FEC electrolyte fails to support the anion-intercalation chemistry on the graphite cathode (Supplementary Fig. 23c). In contrast, the DFEA (both without and with FEC addition) facilitates a reversible anion de-/intercalation process on the graphite host without solvent co-

intercalation (Fig. 1, Supplementary Fig. 5 and Supplementary Fig. 23d). These results indicate that it is the DFEA solvent, rather than the FEC addition, that supports the reversible cathode reaction.

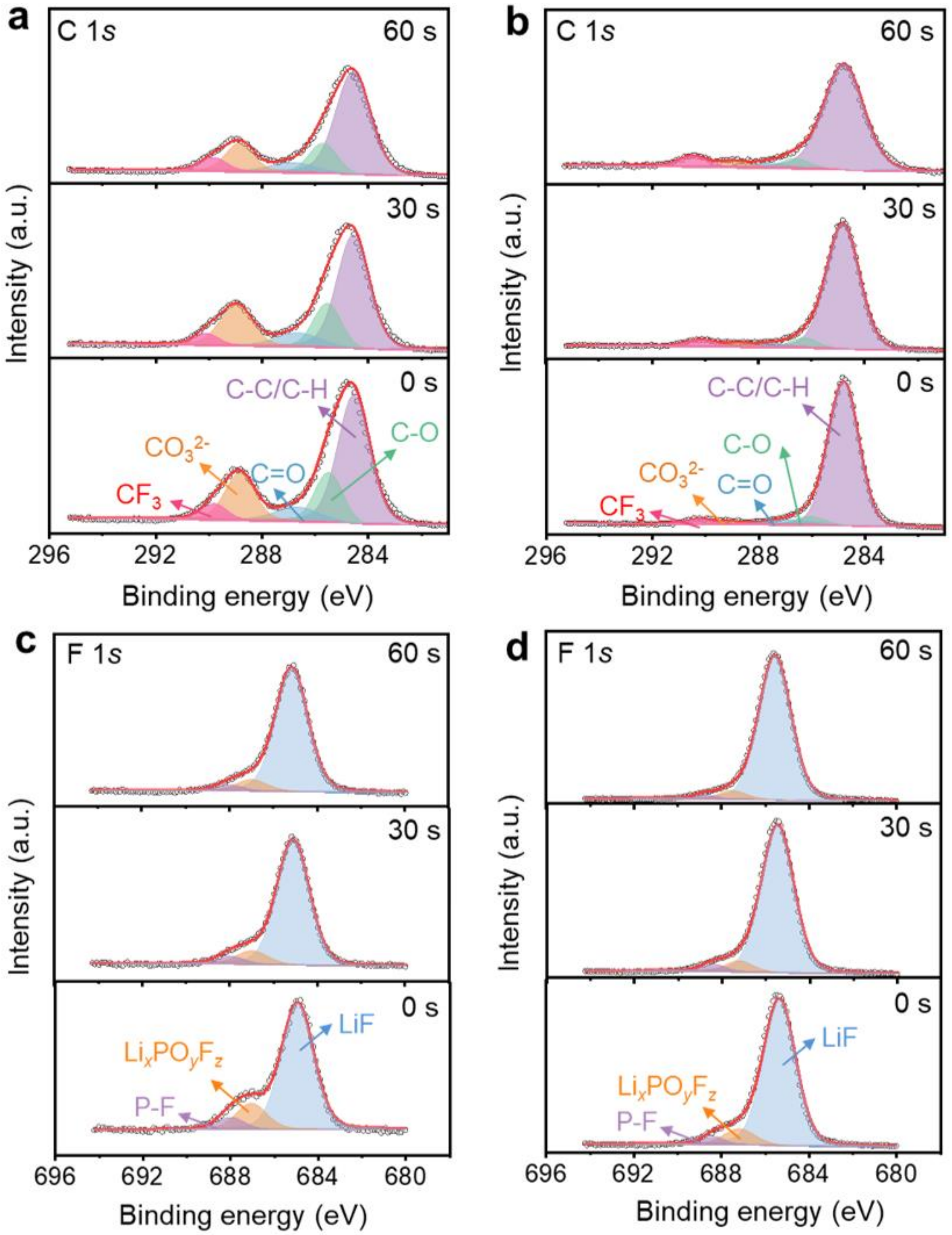

Supplementary Figure 24. F 1*s* XPS depth profiles of the Li metal after using **a** DFEA (without FEC) and **b** DFEA -based electrolytes. LiF: 685 eV; $Li_xPO_yF_z$: 687 eV; CF_3: 688.5 eV.

C 1*s* XPS depth profiles of the Li metal using **c** DFEA (without FEC) and **d** DFEA-based electrolytes. C-C/C-H: 284.5 eV, C-O: 285.5 eV, C=O: 286.7 eV, CO_3^{2-}: 288.8 eV, CF_3: 289.8 eV.

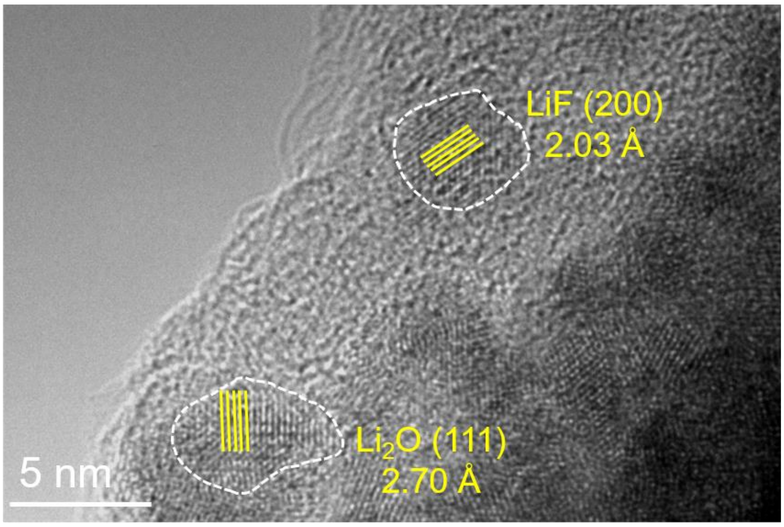

Supplementary Figure 25. TEM image of the SEI shell formed by plating/stripping Li on a Cu grid using DFEA (without FEC) electrolyte.

As displayed in Supplementary Fig. 24 and Supplementary Fig. 25, a large amount of LiF component is observed throughout the whole sputtering process for both electrolytes, indicating that both DFEA (without FEC) and DFEA-based electrolyte contribute to the formation of a LiF-rich SEI layer on the Li metal. It is noted that the FEC addition significantly inhibits the decomposition of both $LiPF_6$ salt and solvents, as evidenced from the larger amounts of $Li_xPO_yF_z$ and organic species (e.g., $ROCO_2Li$) derived from the DFEA (without FEC) electrolyte. In addition, TEM was conducted to investigate the microstructures of SEI shells onto a Cu grid from Li||Cu cells cycled in DFEA (without FEC) electrolyte. As displayed in Supplementary Fig. 25, the lattice spacing of 2.03 Å corresponds to the (200) crystal plane of LiF nanoparticles, indicating that both DFEA (without FEC) and DFEA-based (Fig. 5b) electrolytes lead to the LiF formation on Li anode. However, it is noted that despite the formation of a LiF-enrich SEI with the DFEA (without FEC) electrolyte, the undesired decomposition of the DFEA solvent is not effectively suppressed, as seen from the presence of

Li_2O ((111) plane, 2.70 Å) nanoparticles (Supplementary Fig. 25). These findings are consistent with the XPS results, further proving that both FEC and DFEA solvent contribute to the LiF-rich SEI on the Li metal, while the FEC addition effectively suppress the excessive decomposition of DFEA solvent.

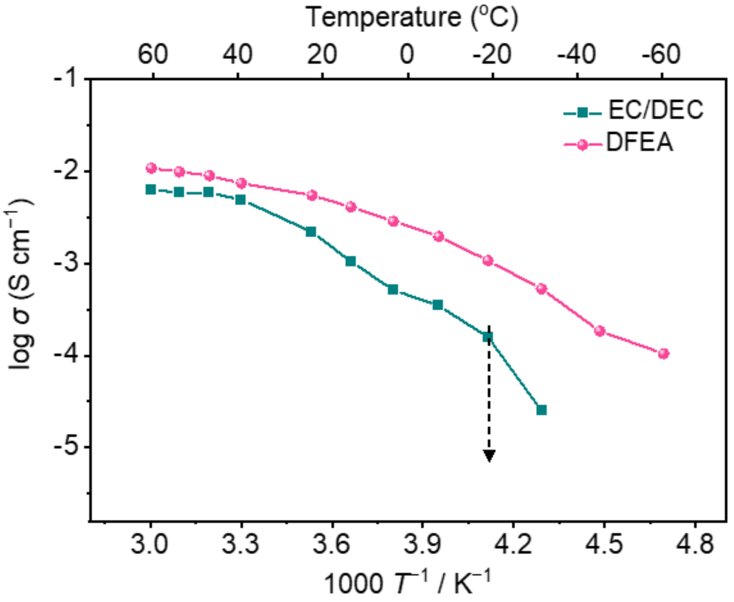

Supplementary Figure 26. Temperature dependence of the ionic conductivities of EC/DEC and DFEA-based electrolytes.

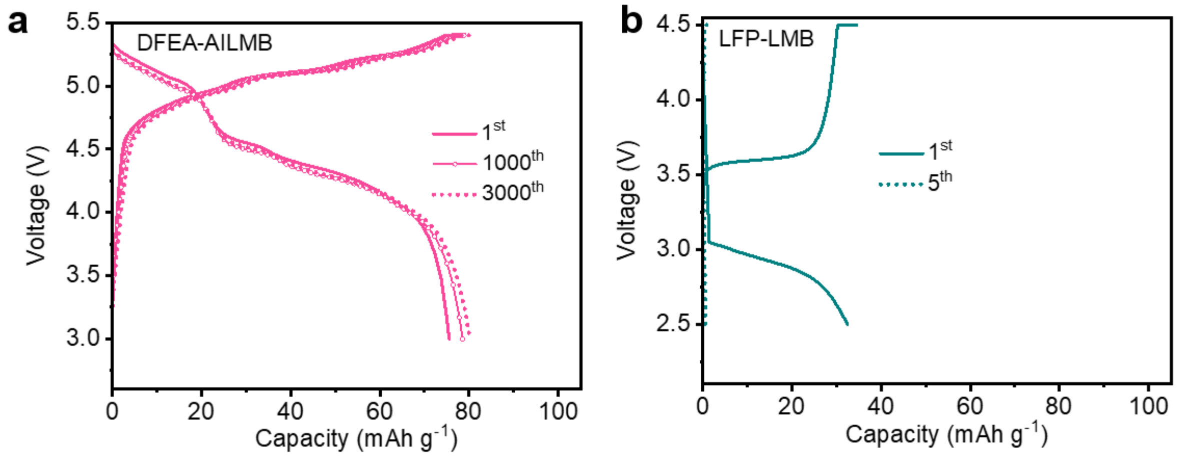

Supplementary Figure 27. Typical charge-discharge curves of **a** AILMBs (using DFEA-based electrolyte) and **b** LFP-LMB (using EC/DEC electrolyte) at −20 °C.

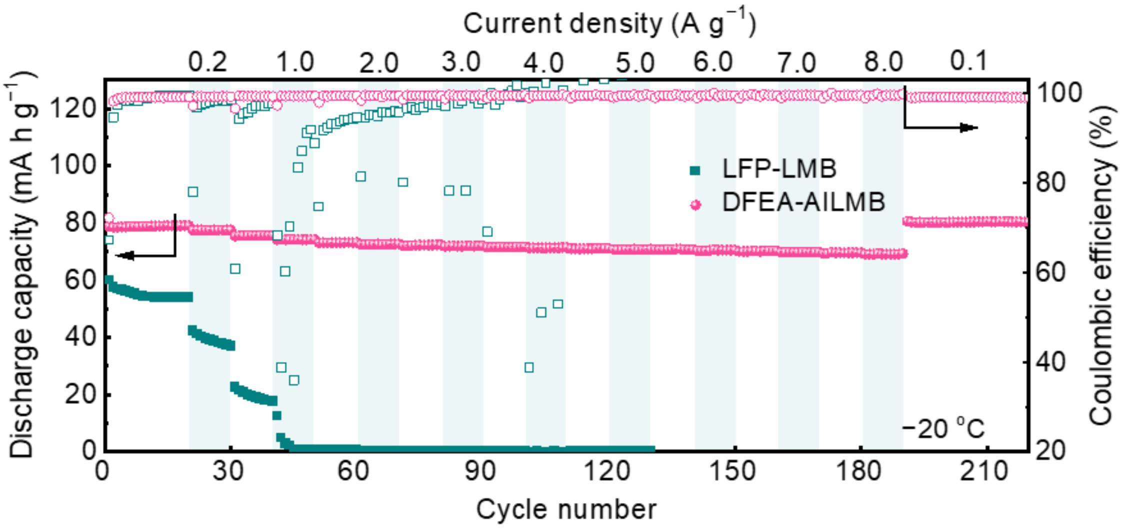

Supplementary Figure 28. Fast-discharging capability of AILMBs (using DFEA-based electrolyte) and LFP-LMB (using EC/DEC electrolyte) at −20 °C, employing CCCV mode for charging process at 100 mg^{-1}, followed by discharging at various current density from 100 mA g^{-1} to 8 A g^{-1}.

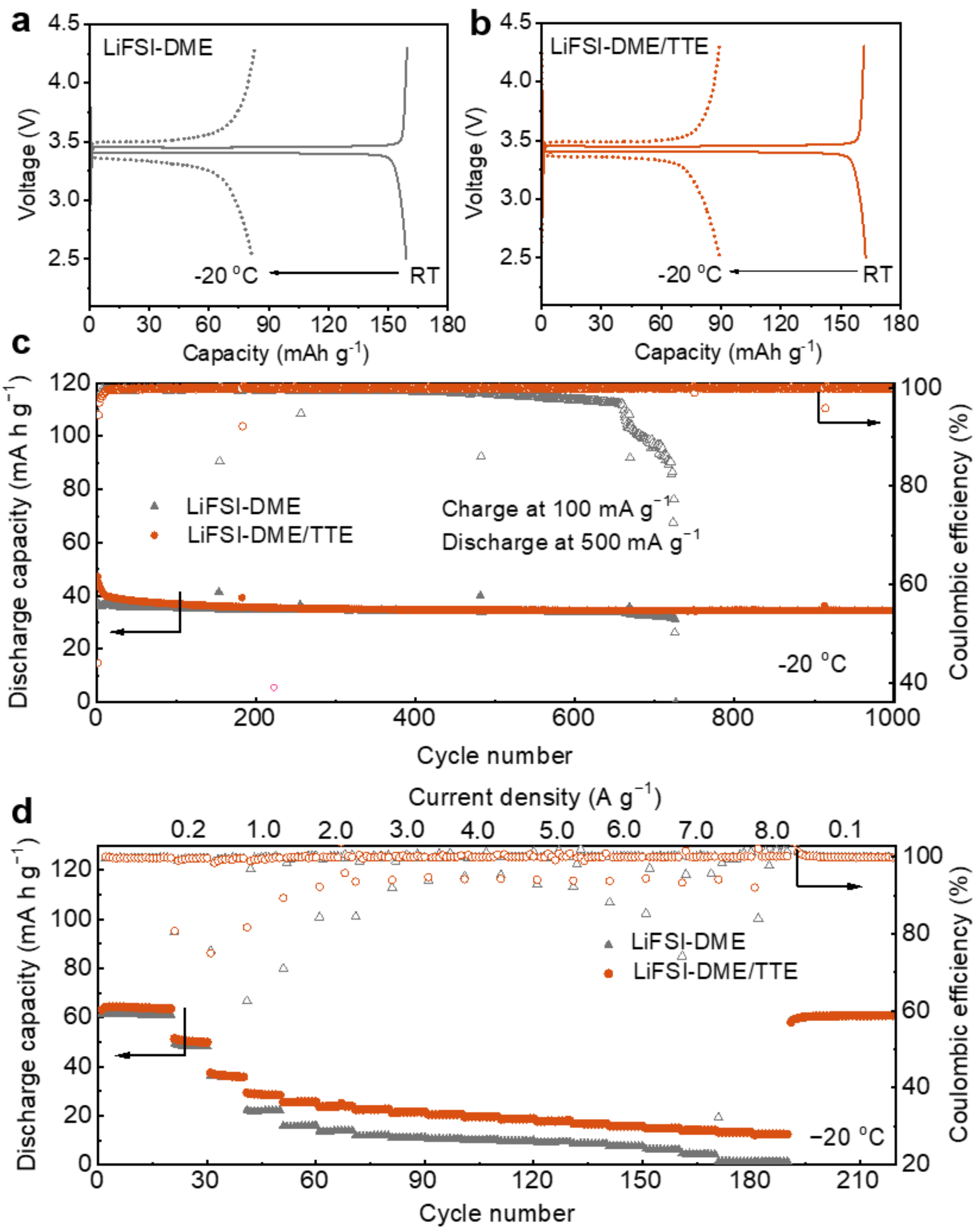

Supplementary Figure 29. **a** Typical charge-discharge profiles of the LFP-LMB using **a** LiFSI-DME and **b** LiFSI-DME/TTE electrolytes at RT and −20 °C. Current density: 20 mA g^{-1}. **c** Long-term cycling performance and **d** fast-discharging capability of the LFP-LMB at −20 °C. Employing CCCV mode for charging process at 100 mA g^{-1}, followed by discharging at various current densities.

Here, the low-temperature performance of Li||LFP cells employing the widely-used Lithium bis(fluorosulfonyl) imide (LiFSI)-dimethyl ether (DME) and LiFSI-DME/1,1,2,2-tetrafluoroethyl-2,2,3,3-tetrafluoropropylether (TTE, 1: 1 by volume) electrolytes was assessed at −20 °C. While the reversible capacities of Li||LFP cells can attain ~82 mAh g^{-1} (using LiFSI-DME, Supplementary Fig. 29a) and ~90 mAh g^{-1} (using LiFSI-DME/TTE, Supplementary Fig. 29b) at a low current density of 20 mA g^{-1}, they suffer from rapid decline at current densities higher than 100 mA g^{-1}. When charging at 100 mA g^{-1} (using CCCV mode) followed by discharging at 500 mA g^{-1}, cells based on these two electrolytes deliver similar reversible capacities of around 40 mAh g^{-1} only, although the TTE co-solvent improves the cell cyclability (Supplementary Fig. 29c). Furthermore, as shown in Supplementary Fig. 29d, the Li||LFP cells exhibit specific capacity as low as only ~22 mAh g^{-1} (using LiFSI-DME) and ~29 mAh g^{-1} (using LiFSI-DME/TTE) at high discharge current density of 1 A g^{-1}.

The low-temperature performance of Li||LFP batteries is significantly degraded at −20 °C, irrespective of the use of commercial carbonate-based electrolytes (Fig. 6b) or prevalent ether-based electrolytes (Supplementary Fig. 29). This can potentially extend the application range of LMBs in low-temperature environments.

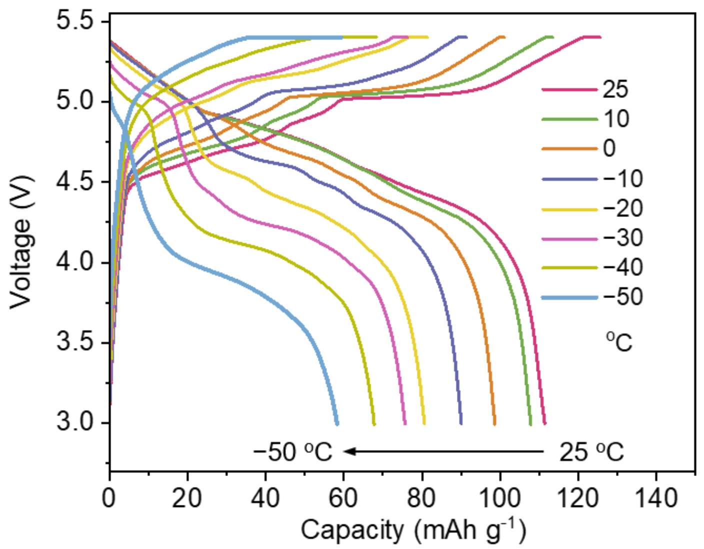

Supplementary Figure 30. Temperature-dependent charge/discharge profiles of AILMBs with DFEA-based electrolyte, adopting the CCCV mode for the charging process at 1 C (25, 10, 0, –10, –20 °C), 0.2 C (–30, –40 °C) and 0.1 C (–50 °C), respectively, followed by discharging at 1 C. 1 C=100 mA g^{-1}.

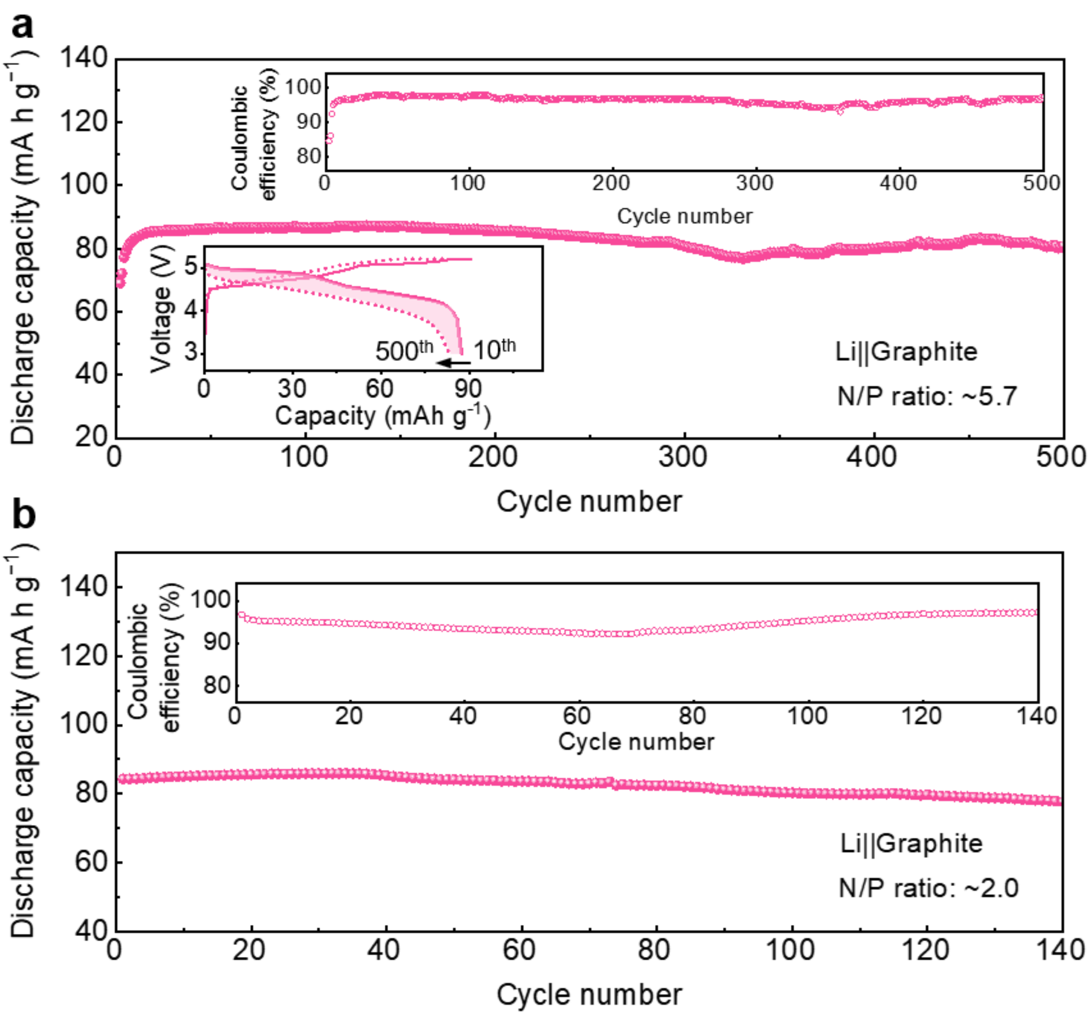

Supplementary Figure 31. Cycling performance of AILMB using DFEA-based electrolyte with a N/P ratio of **a** ~5.7 and **b** ~2.0 at 25 °C, respectively. Inset is the corresponding Coulombic efficiency (upper panels in **a** and **b**) and voltage curves (lower panel in **a**). Employing CCCV mode for charging process at 100 mA g^{-1}, followed by discharging at 100 mA g^{-1}.

We replaced the 450 μm Li metal anode with thin Li foils, reducing the N/P ratio to ~5.7 (with 50 μm-thick Li foil) and ~2.0 (with 20 μm-thick Li foil), respectively (Supplementary Fig. 31). As for the E/C ratio, it is noted that, the slat in electrolyte is considered as active material in AILMBs, thus the amount of electrolyte is inevitably higher than that in conventional LMBs. Considering the E/C ratio is proportional to the electrolyte density (ρ_E) and inversely proportional to the concentration of the electrolyte, the salt concentration has been increased to 3.5 M to minimize the electrolyte weight. A surplus of 20% electrolyte was

used to account for irreversible losses (e.g., SEI and CEI formation) and to guarantee sufficient ionic conductivity at different SOC[6]. Accordingly, a E/C ratio of 2.75 (calculated based on the fully charged state) was used for cell performance assessment. After three activation cycles at 20 mA g^{-1}, the cells were cycled using the CCCV model for charging process (100 mA g^{-1}), followed by discharging at 100 mA g^{-1}.

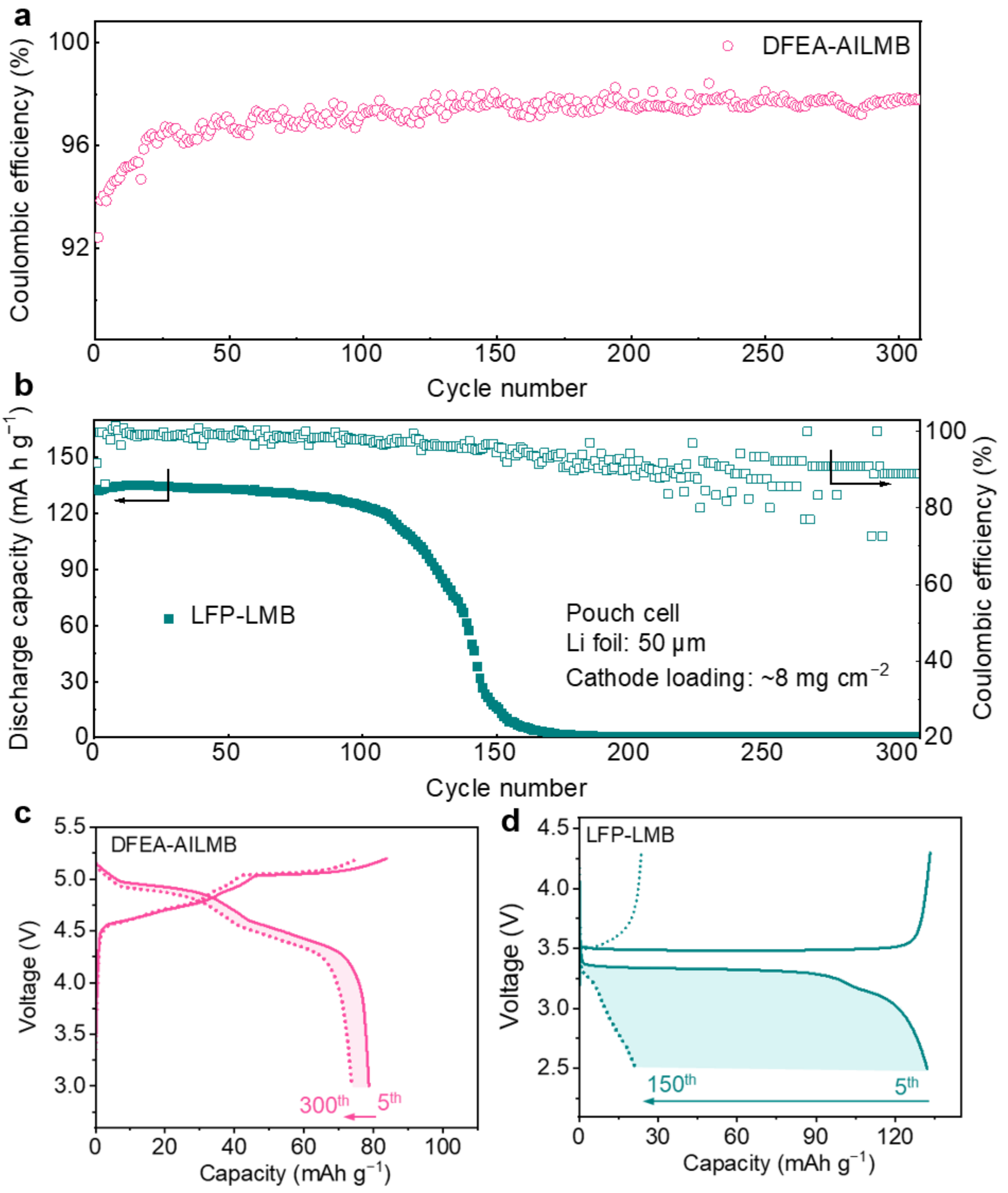

Supplementary Figure 32. a Coulombic efficiency of the 50 μm-thick Li foil||graphite pouch cell at a charge current density of 100 mA g^{-1} and a discharge current density of 100 mA g^{-1}. **b** Cycling performance of the 50 μm-thick Li foil||LFP pouch cells at a charge current density of 100 mA g^{-1} and a discharge current density of 100 mA g^{-1}. **c, d** Corresponding typical charge-discharge curves of **c** Li||graphite (with DFEA-based electrolyte) and **d** Li||LFP (with EC/DEC electrolyte) pouch cells.

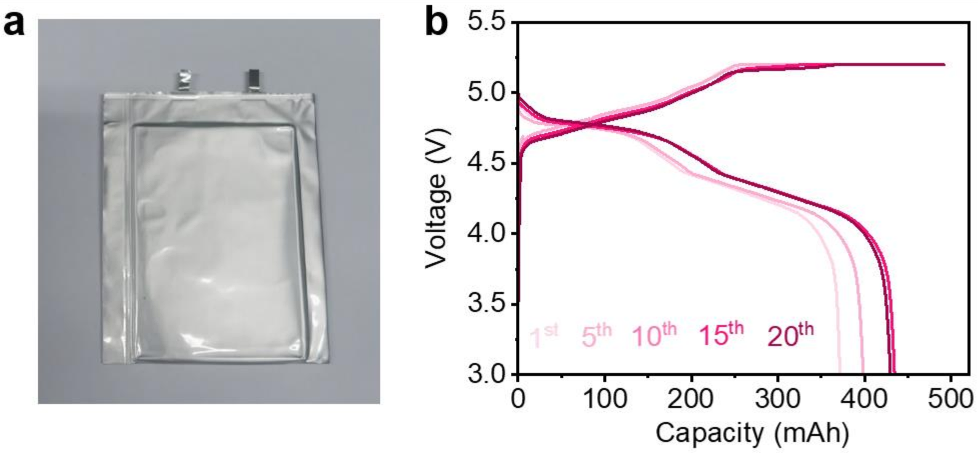

Supplementary Figure 33. **a** Optic image and **b** charge-discharge cycling of the packaged AILMB at 20 mA g^{-1}, 25 ºC.

Supplementary Table 1. Comparisons of the fast-cycling stability of Li||graphite AILMBs with state-of-the-art LMBs.

Cathode material	Electrolyte	Capacity retention /cycle number	Current density (mA g^{-1})	Ref.
NCM811	1 M $LiPF_6$-EC/DEC+ 0.6 wt% LiHMDS	71.24%/1000	180	7
NCM811	1 M $LiPF_6$-EC/DEC +0.1 M $LiNO_3$ +0.1 M LiFEA	83.5%/500	3 mA cm^{-1}	8
LFP	1 M $LiPF_6$-FEC/EC/DEC	97.6%/570	680	9
vertically-aligned-LFP	PEGDME-based polymer electrolyte	74.5%/2000	680	10
Vertically aligned LFP	1 M $LiPF_6$-EC/DEC	86.5%/100	850	11
LFP	ZIF-67-LA-PAM	88.4%/1000	1700	12
poly(pyridinium salt) membrane-coated graphite	2 M $LiPF_6$-EMC	92.8%/1000	100	13
Al_2O_3-coated graphite	4 M $LiPF_6$-EMC	80%/2700	200	14
**Graphite**	**1.2 M $LiPF_6$-DFEA +10 wt% FEC**	**88.0%/10000**	**1000**	**This work**

Supplementary Table 2. Comparisons of the fast-charging capability of reported LMBs. For comparison, the unit of current densities have been standardized to mA cm^{-2}.

Battery configuration	Capacity retention	Current density	Charging time	Ref.
Li|1 M $LiPF_6$-EC/DEC+0.1 M LiTFA+0.1 M $LiNO_3$|NCM811	~67%	6 mA cm^{-2}	6 min	[15]
Li|2 M $LiPF_6$-DMC|C2DP-G	~59.5%	4 mA cm^{-2}	3 min	[16]
Li|1 M $LiPF_6$-EC/DEC+ 0.25 wt.% PFPA+1 wt.% FEC|Mn-rich cathode	~38%	6 mA cm^{-2}	3 min	[17]
Li|TMO|LFP	~50%	~11.9 mA cm^{-2}	3 min	[18]
Li|1 M $LiPF_6$-EC/DEC+1 wt.% BFA|NCM622	~67%	5 mA cm^{-2}	~10 min	[19]
Li||1.2 M $LiPF_6$-EC/DEC|LFP	~52%	7 mA cm^{-2}	~3 min	Performance comparison
**Li|1.2 M $LiPF_6$-DFEA+10 wt.% FEC|Graphite**	**~84%**	16 mA cm^{-2}	**45 s**	**This work**

Supplementary Table 3. The t_{Li}^{+} value of DFEA-based electrolyte (**Supplementary Figure 14**).

R_s^0	Ri^0	R_s^{ss}	R_i^{ss}	I^0	I^{ss}	t_{Li}^{+}
3.364	473.0	3.381	487.7	19.38	17.38	0.49

Supplementary Table 4. Comparisons of the cycling stability of Li||Li cells with reported electrolytes.

Electrolyte	Areal capacity (mAh g^{-1})	Current density (mA g^{-1})	Cycle time (h)	Ref.
2 M LiFSI-TFDMP	1	1	1600	[20]
0.15 M LiFSI+0.15 M LiTFSI+0.15 M $LiNO_3$-DME	1	1	800	[21]
1 m LiFSI-DMTMSA	1.5	0.5	1200	[22]
2 M LiFSI-BFE	1	1	400	[23]
1 M $LiPF_6$-EC/DEC+0.1 M LiTFA+0.1 M $LiNO_3$	3	3	165	[15]
1 M $LiPF_6$-EC/DMC-BFA	2	0.5	330	[19]
0.1 M LiFSI/0.1 M LiTFSI/0.1 M LiDFOB/0.1 M $LiNO_3$/1.0 m $LiPF_6$-EC/DMC, with 5% FEC	0.5	0.5	800	[24]
**1.2 M $LiPF_6$-DFEA+10%FEC**	**0.5**	**0.5**	**3000**	**This work**
**1.2 M $LiPF_6$-DFEA+10%FEC**	**1**	**1**	**1600**	**This work**
**1.2 M $LiPF_6$-DFEA+10%FEC**	**3**	**1**	**930**	**This work**
**1.2 M $LiPF_6$-DFEA+10%FEC**	**4**	**2**	**500**	**This work**

Supplementary Table 5. Fitted values of the impedance spectra in Li||Li cells using EC/DEC and DFEA-based electrolytes (**Supplementary Figure 20**).

T (K)	1.2 M $LiPF_6$-EC/DEC		1.2 M $LiPF_6$-DFEA (FEC additive)	
	R_{sei}	R_{ct}	R_{sei}	R_{ct}
278	787	740.9	32	63.8
288	309.4	283.7	20.8	30.2
298	166	112.4	12.2	14.4
308	52.7	46.0	6.0	8.2
318	22.5	21.3	3.6	5.5

Supplementary Table 6. Calculated specific energy of the packaged AILMB.

**Component**	**Value**
Graphite cathode (96% active material)	5.28 g
Al foil	0.53 g
Li/Cu anode	1.23 g
Electrolyte + Separator	7.14 g
Total weight (exclude packing cell bag), m_{total}	14.18
Reversible capacity, C_{full}	440.5 mAh
Average working voltage, V_{cell}	4.56 V
Specific energy, E_{full}	141.7 Wh kg^{-1}

A 440 mAh Li||graphite multi-layer pouch cell was packaged to determine the specific energy of the AILMB (Supplementary Fig. 33a). The cathode with a high areal capacity of 1.64 mAh cm^{-2} were incorporated to pair with 20 μm-thick Li foil deposited on Cu foils. This configuration gives a low capacity ratio between the anode and cathode (N/P ratio) of ~2.4. As discussed above, to increase the specific energy, the electrolyte amount has to be limited. Here,

the electrolyte concentration has been increased to 3.5 M. Besides, an additional ~10% excess electrolyte was used to ensure sufficient ionic conductivity and to account for irreversible losses during the battery cycling. Electrolyte with a salt concentration of 3.5 M was applied considering the electrolyte amount in AILMBs is inevitably higher than that in conventional LMBs. As shown in Supplementary Table 6, the specific energy of the AILMB using DFEA-based electrolyte is calculated as ~141.7 Wh kg^{-1}.

Supplementary References

1 Jiao, S. et al. Behavior of lithium metal anodes under various capacity utilization and high current density in lithium metal batteries. *Joule* **2**, 110-124 (2018).

2 Cha, J., Han, J.-G., Hwang, J., Cho, J. & Choi, N.-S. Mechanisms for electrochemical performance enhancement by the salt-type electrolyte additive, lithium difluoro(oxalato)borate, in high-voltage lithium-ion batteries. *J. Power Sources* **357**, 97-106 (2017).

3 Kuwata, H., Sonoki, H., Matsui, M., Matsuda, Y. & Imanishi, N. Surface layer and morphology of lithium metal electrodes. *Electrochemistry* **84**, 854-860 (2016).

4 Yu, Z. *et al.* Rational solvent molecule tuning for high-performance lithium metal battery electrolytes. *Nat. Energy* **7**, 94-106 (2022).

5 Abraham, K. M., Jiang, Z. & Carroll, B. Highly conductive PEO-like polymer electrolytes. *Chem. Mater.* **9**, 1978-1988 (1997).

6 Placke, T. *et al.* Perspective on performance, cost, and technical challenges for practical dual-ion batteries. *Joule* **2**, 2528-2550 (2018).

7 Zhang, D. *et al.* Lithium hexamethyldisilazide as electrolyte additive for efficient cycling of high-voltage non-aqueous lithium metal batteries. *Nat. Commun.* **13**, 6966 (2022).

8 Xia, Y. *et al.* Designing an asymmetric ether-like lithium salt to enable fast-cycling high-energy lithium metal batteries. *Nat. Energy* **8**, 934-945 (2023).

9 Yang, T. *et al.* From flower-like to spherical deposition: a GCNT aerogel scaffold for fast-charging lithium metal batteries. *Adv. Energy Mater.* **11**, 2102454 (2021).

10 Gao, X. *et al.* Fast charging all solid-state lithium batteries enabled by rational design of dual vertically-aligned electrodes. *Adv. Funct. Mater.* **30**, 2005357 (2020).

11 Guo, Y., Jiang, Y., Zhang, Q., Wan, D. & Huang, C. Directional $LiFePO_4$ cathode structure by freeze tape casting to improve lithium ion diffusion kinetics. *J. Power Sources* **506**, 230052 (2021).

12 Guan, J. *et al.* A new in situ prepared MOF-natural polymer composite electrolyte for solid lithium metal batteries with superior high-rate capability and long-term cycling stability at ultrahigh current density. *Adv. Sci.* **10**, 2203916. (2023).

13 Sabaghi, D. *et al.* Ultrathin positively charged electrode skin for durable anion-intercalation battery chemistries. *Nat. Commun.* **14**, 760 (2023).

14 Li, W. H. *et al.* All-climate and ultrastable dual-ion batteries with long life achieved via synergistic enhancement of cathode and anode interfaces. *Adv. Funct. Mater.* **32**, 2201038 (2022).

15 Xia, Y. *et al.* Designing an asymmetric ether-like lithium salt to enable fast-cycling high-energy lithium metal batteries. *Nat. Energy* **8**, 934-945 (2023).

16 Sabaghi, D. *et al.* Ultrathin positively charged electrode skin for durable anion-intercalation battery chemistries. *Nat. Commun.* **14**, 760 (2023).

17 An, K. *et al.* Ultrafast Charging of a 4.8 V Manganese-rich cathode-based lithium metal cell by constructing robust solid electrolyte interphases. *Adv. Funct. Mater.* **33**, 2301755 (2023).

18 Wang, H. *et al.* A fluoride-rich solid-like electrolyte stabilizing lithium metal batteries. *Adv. Mater.*, 2313135 (2024).

19 Li, F. *et al.* Gradient solid electrolyte interphase and lithium-ion solvation regulated by bisfluoroacetamide for stable lithium metal batteries. *Angew. Chem. Int. Ed.* **60**, 6600-6608 (2021).

20 Zhao, Y., Zhou, T., Mensi, M., Choi, J. W. & Coskun, A. Electrolyte engineering via ether solvent fluorination for developing stable non-aqueous lithium metal batteries. *Nat. Commun.* **14**, 299 (2023).

21 Wang, Q. *et al.* High entropy liquid electrolytes for lithium batteries. *Nat. Commun.* **14**, 440 (2023).

22 Xue, W. *et al.* Ultra-high-voltage Ni-rich layered cathodes in practical Li metal batteries enabled by a sulfonamide-based electrolyte. *Nat. Energy* **6**, 495-505 (2021).
23 Zhang, G. *et al.* A monofluoride ether-based electrolyte solution for fast-charging and low-temperature non-aqueous lithium metal batteries. *Nat. Commun.* **14**, 1081 (2023).
24 Wang, Q. *et al.* Entropy-driven liquid electrolytes for lithium batteries. *Adv. Mater.* **35**, 2210677 (2023).
